# Changes in blood pressure, glucose levels, insulin secretion and anthropometry after long term exposure to antiretroviral therapy in South African women

**DOI:** 10.1186/s12981-015-0065-8

**Published:** 2015-08-05

**Authors:** Zulfa Abrahams, Joel A Dave, Gary Maartens, Naomi S Levitt

**Affiliations:** Division of Diabetic Medicine and Endocrinology, Department of Medicine, University of Cape Town, Cape Town, South Africa; Division of Clinical Pharmacology, Department of Medicine, University of Cape Town, Cape Town, South Africa

**Keywords:** HIV, Dysglycaemia, Hypertension, Antiretroviral therapy, Body composition, Lipoatrophy, Blood pressure

## Abstract

**Background:**

A number of metabolic abnormalities, such as dysglycaemia, insulin resistance, lipodystrophy and dyslipidaemia, are associated with the use of antiretroviral drugs. We aimed to assess the effects of long-term antiretroviral exposure on blood pressure, glycaemia, insulin secretion and anthropometric measures in black South African women.

**Methods:**

A convenience sample of HIV-infected women on first-line ART for a median of 16 months at baseline, had the following evaluations twice, at baseline and after approximately 5 years: anthropometry, including skin fold thicknesses, blood pressure, oral glucose test, and insulin. Insulin sensitivity and secretion (HOMA-IR, IGI and DI_o_) were estimated.

**Results:**

At baseline more than half the 103 women were using stavudine and efavirenz. The median interval between baseline and follow-up evaluation was 66 months. Weight, waist circumference, and waist-hip ratio increased over time, while limb skinfold thickness decreased over time. Systolic and diastolic blood pressure increased significantly and the proportion of participants with hypertension increased from 3.9 to 15.5% (p < 0.001). There were increases from baseline in plasma glucose concentrations at 30 and 120 min; insulin concentrations at 0 and 30 min; and IGI and DI_o_. The proportion of participants with diabetes increased from 1 to 7.5% (p = 0.070).

**Conclusion:**

In black South African women with long-term exposure to ART, increases in hypertension and possibly diabetes were observed. Participants experienced an increase in central fat and a decrease in peripheral fat distribution. Early identification and management of these metabolic changes are important, especially in a region with the highest HIV-infected population in the world.

## Background

Africa has made great strides in expanding access to antiretroviral therapy (ART), with an estimated 7.6 million people in sub-Saharan Africa receiving treatment by December 2012 [1]. The increase in access to ART has resulted in a dramatic decline in HIV-related deaths. However, several antiretroviral drugs are associated with a number of metabolic abnormalities [2] including dyslipidaemia, lipodystrophy, insulin resistance and dysglycemia [3].

Several studies from Africa have shown an increased prevalence of dysglycaemia in HIV-infected patients, especially in patients on ART, but the duration of ART exposure was generally under 3 years [4–6]. There are conflicting data with regard to the impact of HIV and ART on hypertension, with some studies showing an increased risk of hypertension [7, 8] and others showing no association [9–11]. A recent systematic review [12] found that HIV-infected patients in sub-Saharan Africa, irrespective of ART status, had lower systolic and diastolic blood pressure (BP) than HIV-uninfected controls.

ART-related lipoatrophy is common in low-and-middle income countries (LMICs) [13–15], where stavudine has only recently been phased out and zidovudine is still widely being used. Anthropometric studies show that fat loss is best detected by triceps skinfold and hip circumference measurements [13, 16]. Central fat accumulation on ART is thought to be a consequence of treating the HIV infection, as the gain in trunk and visceral fat is no different between HIV-infected participants on ART and HIV-uninfected controls, and does not differ by antiretroviral class [17].

Little is known about the long-term metabolic effects of ART in LMICs. We aimed to assess the effects of long-term ART exposure on blood pressure, glycaemia, insulin secretion and anthropometric measures in black South African women.

## Results

Participant characteristics are presented in Table 1. We enrolled 103 of the 345 women assessed at baseline for follow-up assessment. At baseline the participants had spent a median of 16 months on first-line ART and almost all were using stavudine (91%) and lamivudine (100%). At follow-up 84% were still on first-line ART but the percentage of those using stavudine had decreased to 39%, and those using zidovudine had increased from 10 to 38%. The median time on ART at follow-up was 82 months (6.8 years).

**Table 1:** Comparison of baseline and follow-up characteristics of female participants

	Baseline median (IQR), n = 84	Follow-up median (IQR), n = 77
Age	33.5 (30.0–40.0)	40.1 (35.7–45.4)
Current CD4 count	372 (261–471)	564 (427–774)
Time on ART (months)	16.0 (10.0–26.0)	82.4 (73.8–94.1)

	n (%)	n (%)
No schooling	5 (4.8)	
Primary School	14 (13.6)	
Secondary School	83 (80.6)	
Tertiary	1 (1.0)	
1st line ART	84 (100)	65 (84.4)
2nd line ART	0 (0)	12 (15.6)
Stavudine	76 (90.5)	30 (39.0)
Lamivudine	84 (100)	77 (100)
Zidovudine	8 (9.5)	29 (37.7)
Tenofovir	0 (0.0)	18 (23.4)
Lopinavir	0 (0.0)	12 (15.6)
Efavirenz	41 (48.8)	36 (46.8)
Nevirapine	43 (51.2)	29 (37.7)

As shown in Table 2, waist circumferences increased significantly (p = 0.038), while hip and mid-thigh circumferences decreased (p < 0.001). All skinfold thicknesses changed significantly from baseline to follow-up. All 25% of the participants who reported having lipoatrophy at baseline, reported none at follow-up. However, when using objective measures [13] based on thigh and triceps cut points, the percentage of participants with lipoatrophy increased from 44 to 64%; p = 0.010. At baseline, 60% of those who self-reported moderate or severe fat loss in 2 or more regions, were correctly classified using thigh and tricep skinfold cut points, while 61.5% of those who reported having lost none, or minimal amounts of fat were correctly classified (p = 0.002).

**Table 2:** Comparison of anthropometric measures in females at baseline and follow-up (n = 103)

	Baseline median (IQR), n = 103	Follow-up median (IQR) n = 94	P-value*
Height (m)	1.6 (1.5–1.6)	1.6 (1.5–1.6)	0.401
Weight (kg)	69.2 (61.4–81.1)	70.1 (59.7–78.8)	0.402
BMI	27.9 (24.8–31.8)	27.8 (23.9–31.6)	0.443
Sagittal height (cm)	21 (19–24)	20.6 (18.5–23.5)	0.640
Circumferences			
Waist (cm)	89.8 (81.3–96.8)	90.8 (82.5–100.0)	0.038
Hip (cm)	103.0 (96.0–114.0)	100.0 (91.8–106.5)	<0.001
Waist-hip ratio	0.86 (0.81–0.92)	0.92 (0.85–0.98)	<0.001
Mid-upper arm (cm)	29.0 (27.0–32.0)	29.5 (26.8–32.5)	0.292
Mid-thigh (cm)	58.0 (53.0–63.0)	55.0 (49.5–59.5)	<0.001
Skinfold thickness			
Biceps (mm)	8.1 (5.8–10.5)	9.0 (6.4–12.6)	0.011
Triceps (mm)	19.0 (12.6–25.2)	16.3 (11.3–22.5)	0.007
Abdomen (mm)	25.1 (16.9–34.2)	32.2 (21.7–37.8]	<0.001
Thigh (mm)	32.8 (24.1–43.3)	24.3 (17.3–34.0)	<0.001
Sub-scapular (mm)	21.5 (13.4–28.8)	29.0 (18.8–34.2)	<0.001
Supra-iliac (mm)	16.1 (9.9–22.4)	20.5 (13.3–28.9]	<0.001
Calf (mm)	17.8 (12.3–24.4)	13.1 (7.0–19.2)	<0.001

	n (%)	n (%)	P-value**
Lipoatrophy			
Based on patient report	25 (24.5)	0	<0.001
Based on thigh (≤28 mm) and triceps (≤14.5 mm) skinfold cut points	45 (43.7)	59 (64.1)	0.010

* Non-parametric paired t-test.

** McNemar Chi square test for paired data.

Both systolic and diastolic blood pressures increased (p < 0.001) from baseline to follow-up (Table 3). Plasma glucose concentrations at 30 and 120 min, and insulin concentrations at 0 and 30 min also increased significantly from baseline to follow-up (p < 0.050). Although the homeostatic assessment model (HOMA-IR) tended to increase (p = 0.089) from baseline to follow-up, both the insulinogenic index (IGI) and the oral disposition index (DI_o_) increased significantly (p < 0.001).

**Table 3:** Comparison of blood pressure, plasma glucose and insulin concentrations and markers of insulin sensitivity and beta cell function at baseline and follow-up (n = 103)

	Baseline median (IQR)	Follow-up median (IQR)	P-value*
Blood Pressure			
Systolic	111 (101–121)	121 (112–133)	<0.001
Diastolic	72 (64–80)	80 (73–89)	<0.001
Glucose			
Fasting	5.1 (4.7–5.4)	4.9 (4.7–5.3)	0.365
30 min	6.6 (5.8–7.4)	6.8 (5.9–8.0)	0.040
120 min	5.4 (4.9–6.3)	5.6 (4.7–6.8)	0.028
Insulin			
Fasting	5.6 (3.3–9.5)	7.9 (4.1–12.9)	0.009
30 min	35.1 (19.2–66.7)	177.7 (163.4–192.5)	<0.001
120 min	24.0 (13.5–40.2)	23.7 (10.4–54.9)	0.993
Glycaemic parameters (without diabetics and outliers)			
HOMA-IR^a^	1.2 (0.7–2.2)	1.6 (0.9–2.7)	0.089
IGI^b^	23.7 (11.9–33.1)	80.0 (54.0–137.4)	<0.001
DI_o_^c^	3.5 (2.3–7.9)	11.9 (4.9–23.3)	<0.001

* Non-parametric paired t-test.

^a^HOMA-IR = (fasting glucose × fasting insulin)/22.5.

^b^IGI = ΔInsulin_0-30_/ΔGlucose_0-30_.

^c^DI_o_ [Oral disposition index] = (ΔInsulin_0-30_/ΔGlucose_0-30_) × (1/fasting insulin).

The proportion of participants with hypertension increased from baseline to follow-up, from 3.9 to 15.5%; p < 0.001 (Table 4). While the proportion of participants with impaired glucose tolerance (IGT), impaired fasting glucose (IFG) and dysglycemia did not change significantly from baseline to follow-up, there was a trend to an increase in the proportion of participants with diabetes (1% to 7.5%; p = 0.070). At baseline and follow-up, diabetes, hypertension and dysglycaemia were significantly associated with lipoatrophy (p < 0.001) based on thigh and tricep skinfold cut points.

**Table 4:** Comparison of blood pressure and glucose abnormalities in females at baseline and follow-up (n = 103)

	Baseline n (%)	Follow-up n (%)	P-value*
Hypertension	3 (3.9)	16 (15.5)	<0.001
Glucose abnormalities^a^			
Diabetes	1 (1.0)	7 (7.5)	0.070
Impaired glucose tolerance	6 (5.8)	9 (9.6)	0.344
Impaired fasting Glucose	17 (16.5)	10 (10.5)	0.308
Dysglycemia	22 (21.4)	19 (20)	1.00

* McNemar Chi square test for paired data.

^a^n = 94 at follow-up.

Stavudine, efavirenz and nevirapine were significantly associated with diabetes, hypertension and dysglycaemia at follow-up (Table 5). Lipoatrophy was significantly associated (p < 0.001) with zidovudine, tenofovir, lopinavir in addition to stavudine, efavirenz and nevirapine.

**Table 5:** P-values* representing associations between diabetes, hypertension, dysglycaemia and lipoatrophy, and different antiretroviral drugs at follow-up

	Diabetes	Hypertension	Dysglycaemia	Lipoatrophy^a^
Stavudine	0.001	0.017	0.029	<0.001
Zidovudine	0.031	1	0.845	<0.001
Tenofovir	0.004	0.690	0.856	<0.001
Lopinavir	0.077	0.664	0.690	<0.001
Efavirenz	0.001	<0.001	<0.001	<0.001
Nevirapine	<0.001	0.029	0.029	<0.001

* McNemar Chi square test for paired data.

^a^Defined by thigh and tricep skinfold cut points.

## Discussion

Our results show that long term exposure to ART in South African women is associated with increases in blood pressure, glucose and insulin levels. These women also experienced changes in body composition with a significant increase in the waist-hip ratio, and in the prevalence of lipoatrophy when objective anthropometric measures (thigh and triceps skinfold cut points [13]) were used instead of the subjective measure of patient report. These metabolic and body composition changes are all associated with an increased cardiovascular risk [18].

The prevalence of hypertension at baseline in our study was threefold lower than in women of a similar age-group from a similar area in Cape Town who participated in a community-based cardiovascular risk factor study (CRIBSA). At follow-up the prevalence of hypertension was lower when compared to similarly aged women from the CRIBSA Study. Although HIV testing was not performed in the CRIBSA Study, participants were not known to be on ART and based on local data, the HIV-infected proportion was estimated to be about 10% [19]. Another study from rural KwaZulu Natal, South Africa [20] reported a 20% prevalence of hypertension in HIV-infected women compared to 40% in HIV-uninfected women 15 years and older. The lower BMI in people on ART compared to the HIV negative participants may be an explanation for the lower prevalence of hypertension in both of these South African studies. In contrast, studies from Tanzania [7] and Uganda [7, 21] have reported a similar prevalence of hypertension in HIV-infected and HIV-uninfected participants, with those on ART in Tanzania having a higher BMI than those who were HIV-uninfected or HIV-infected and ART-naive.

Although the prevalence of new onset diabetes increased between baseline and follow-up in our study, this did not reach statistical significance, possibly due to the small sample size. Interestingly, the prevalence of new onset diabetes at follow-up, was twofold higher in our study than the prevalence of new onset diabetes in women of similar ages from the CRIBSA study [19]. The different methods used to assess dysglycaemia makes it difficult to compare studies from Africa. However, a study [22] that also used an OGTT to assess dysglycaemia found a similar prevalence of dysglycaemia, although the length of time on ART was longer in our study (81 months vs 48 weeks). The rise in insulin secretion in relation to insulin resistance, as expressed by the DI_o_, in the majority of the group at follow-up explains their lack of development of diabetes.

Although there was no increase in BMI at follow-up, the greater abdominal skinfold thickness and waist-hip ratio together with peripheral wasting suggests a marked difference in body composition with centralisation of body fat, in agreement with a number of other African studies [5, 13, 16, 23]. HIV-associated central fat accumulation likely reflects the consequence of treating the HIV infection rather than a specific antiretroviral adverse drug reaction [17]. We found an increase in the percentage of participants with lipoatrophy when defined by thigh and triceps skinfold cut points [13]. However, when using patient report to diagnose lipoatrophy, no women had lipoatrophy, including the 25% who reported lipoatrophy at baseline. The discrepancy we found in the proportion of women with lipoatrophy on anthropometry and patient report is likely due to the women having grown accustomed to their new body shape and illustrates the limitations of diagnosing lipodystrophy using a subjective measure. Lipoatrophy is an antiretroviral adverse drug reaction, strongly associated with the use of thymidine analogue nucleoside reverse transcriptase inhibitors (NRTI’s), stavudine and zidovudine [17]. In our study more than 30% of women were still on stavudine at follow-up and almost 20% were still taking zidovudine. The baseline prevalence of lipoatrophy we found is also similar to that of another South African study [16], which reported a 43% prevalence after 2 years of treatment, but they defined lipoatrophy only by subjective patient and healthcare worker reports.

Our study has some limitations. The lack of a HIV-uninfected and ART-naïve control groups limits our ability to attribute the changes observed to the use of ART. The sample size was also relatively small, which limited our ability to assess whether the increased prevalence of diabetes over time was significant. Despite these limitations, ours is one of very few studies in Africa to use an OGTT to define dysglycemia and to follow women on ART for over 5 years.

## Conclusion

In this study from Africa we observed that women who had been on ART for more than 5 years, developed increased blood pressure (systolic and diastolic). The prevalence of hypertension and diabetes also increased. The greatest changes observed were in body composition, with an increase in central fat and a decrease in subcutaneous fat. The prevalence of lipoatrophy, when defined by skinfold cut points, increased substantially. These findings have important implications for the management of HIV in Africa. The early identification and management of these cardiometabolic risks are crucial in the region with the highest HIV-infected population in the world.

## Methods

### Participants

In our initial cross sectional study, undertaken in 2007–2008 to examine the metabolic consequences of ART, a convenience sample of 345 HIV-infected black African women on first-line ART who were being followed up at ART clinics in Cape Town were selected. The recruitment procedure is described elsewhere [5]. At that stage the first-line ART regimen consisted of stavudine, lamivudine or zidovudine and efavirenz or nevirapine, and the second-line regimen consisted of zidovudine with didanosine and lopinavir/ritonavir [24]. Subsequently, the first-line regimen was changed to tenofovir and efavirenz or nevirapine, and lamivudine replaced didanosine in the second-line regimen. NRTI drug substitutions for toxicity or the convenience of a fixed dose combination are not considered switches to 2nd line ART. 103 of the initial 345 participants could be traced approximately 5 years later and underwent repeated assessments. The remainder could not be traced using their home address or telephone number and were no longer attending the health facility from which they were recruited; defaulted, were pregnant or had died. The baseline characteristics did not differ between those we traced and those not traced.

### Testing procedures

We used the same procedures to collect information from participants at baseline and follow-up. Socio-demographic information was collected using an interviewer administered questionnaire. Clinical records were obtained from health facilities and reviewed to obtain data on ART regimen, time on ART and, CD4 count. The Lipodystrophy Case Definition questionnaire [25] was used to collect self-reported information on fat gain or fat loss. Self-reported lipoatrophy was defined as in the HOPS study, as moderate or severe fat loss in 2 or more regions and self-reported lipohypertrophy defined as moderate or severe fat gain in two or more areas [26].

After an overnight fast, participants underwent a 75 g oral glucose tolerance test (OGTT). Venous blood samples were taken at 0, 30 and 120 min. The plasma was stored and analysed as previously described [5].

Anthropometric measurements: [weight, height, circumferences (waist, hip, mid-upper arm, and mid-thigh), skinfold thickness (biceps, triceps, subscapular, abdomen, suprailiac, thigh and calf) and sagittal abdominal diameter (SAD)] were also done. Cut point based lipoatrophy was defined as having a thigh skinfold thickness ≤28 mm or a triceps skinfold thickness of ≤14.5 mm [13]. Three BP measurements were taken at 2-min intervals using an Omron BP monitor with an appropriately sized cuff after the participant had been seated for 5 min. The average of the second and third BP measurements was used in the analyses. Hypertension was defined as BP ≥140/90 mmHg or using antihypertensive agents. Diabetes, IGT and IFG were defined using the American Diabetes Association criteria [27].

### Ethical approval

The study proposal was submitted and approved by the Research Ethics Committee of the Faculty of Health Sciences at the University of Cape Town. Written informed consent was obtained from all participants prior to participation in the study, at baseline and again at follow-up.

### Data analyses

Data analysis was carried out using the STATA/SE statistical software package version 12.0 (StataCorp., College Station, TX, USA). Baseline data were collected between February 2007 and June 2009 and follow-up data between July 2011 and July 2013. Because the data were not normally distributed, continuous variables were described as medians and inter-quartile ranges (IQR), and were compared using a non-parametric paired *t* test. Binary variables were described using numbers and percentages, and compared using the McNemar Chi square test for paired data.

Markers of beta cell function and insulin resistance were estimated in the participants who did not have diabetes at follow-up. Beta-cell function was estimated using (1) IGI, calculated as the ratio of the change in insulin to the change in glucose from 0 to 30 min (ΔInsulin_0–30_/ΔGlucose_0–30_), and (2) DI_o_, calculated as a (ΔInsulin_0-30_/ΔGlucose_0–30_) × (1/fasting insulin). Insulin resistance was estimated using HOMA-IR, calculated as (fasting glucose × fasting insulin)/22.5.

## References

[1] UNAIDS: Access to antiretroviral therpy in Africa—status report on progress towards 2015 targets. 2013. http://www.unaids.org/sites/default/files/media_asset/20131219_AccessARTAfricaStatusReportProgresstowards2015Targets_en_0.pdf. Accessed 23 April 2015

[2] Schambelan M, Benson CA, Carr A, Currier JS, Dubé MP, Gerber JG (2002). Management of metabolic complications associated with antiretroviral therapy for HIV-1 infection: recommendations of an International AIDS Society-USA panel. J Acquir Immune Defic Syndr.

[3] Gutierrez AD, Balasubramanyam A (2012). Dysregulation of glucose metabolism in HIV patients: epidemiology, mechanisms, and management. Endocrine.

[4] Mutimura E, Stewart A, Rheeder P, Crowther NJ (2007). Metabolic function and the prevalence of lipodystrophy in a population of HIV-infected African subjects receiving highly active antiretroviral therapy. J Acquir Immune Defic Syndr.

[5] Dave JA, Lambert EV, Badri M, West S, Maartens G, Levitt NS (2011). Effect of nonnucleoside reverse transcriptase inhibitor-based antiretroviral therapy on dysglycemia and insulin sensitivity in South African HIV-infected patients. J Acquir Immune Defic Syndr.

[6] Omech B, Sempa J, Castelnuovo B, Opio K, Otim M, Mayanja-Kizza et al (2012) Prevalence of HIV-associated metabolic abnormalities among patients taking first-line antiretroviral therapy in Uganda. ISRN AIDS. doi:10.5402/2012/960178 10.5402/2012/960178 PMC3767451 24052885

[7] Peck RN, Shedafa R, Kalluvya S, Downs JA, Todd J, Suthanthiran M (2014). Hypertension, kidney disease, HIV and antiretroviral therapy among Tanzanian adults: a cross-sectional study. BMC Med.

[8] Gazzaruso C, Bruno R, Garzaniti A, Giordanetti S, Fratino P, Sacchi P (2003). Hypertension among HIV patients: prevalence and relationships to insulin resistance and metabolic syndrome. J Hypertens.

[9] Ogunmola OJ, Oladosu OY, Olamoyegun AM (2014). Association of hypertension and obesity with HIV and antiretroviral therapy in a rural tertiary health center in Nigeria: a cross-sectional cohort study. Vasc Health Risk Manag.

[10] Bergersen B, Sandvik L, Dunlop O, Birkeland K, Bruun J (2003). Prevalence of hypertension in HIV-positive patients on highly active retroviral therapy (HAART) compared with HAART-naive and HIV-negative controls: results from a Norwegian study of 721 patients. Eur J Clin Microbiol Infect Dis.

[11] Jerico C, Knobel H, Montero M, Sorli ML, Guelar A, Gimeno JL (2005). Hypertension in HIV-infected patients: prevalence and related factors. Am J Hypertens.

[12] Dillon DG, Gurdasani D, Riha J, Ekoru K, Asiki G, Mayanja BN, Levitt NS (2013). Association of HIV and ART with cardiometabolic traits in sub-Saharan Africa: a systematic review and meta-analysis. Int J Epidemiol.

[13] Abrahams Z, Dave JA, Maartens G, Lesosky M, Levitt NS (2014). The development of simple anthropometric measures to diagnose antiretroviral therapy-associated lipodystrophy in resource limited settings. AIDS Res Ther.

[14] Menezes C, Maskew M, Sanne I, Crowther N, Raal F (2011). A longitudinal study of stavudine-associated toxicities in a large cohort of South African HIV infected subjects. BMC Infect Dis.

[15] van Griensven J, De Naeyer L, Mushi T, Ubarijoro S, Gashumba D, Gazille C (2007). High prevalence of lipoatrophy among patients on stavudine-containing first-line antiretroviral therapy regimens in Rwanda. Trans R Soc Trop Med Hyg.

[16] George JA, Venter WD, Van Deventer HE, Crowther NJ (2009). A longitudinal study of the changes in body fat and metabolic parameters in a South African population of HIV-positive patients receiving an antiretroviral therapeutic regimen containing stavudine. AIDS Res Hum Retroviruses.

[17] de Waal R, Cohen K, Maartens G (2013). Systematic review of antiretroviral-associated lipodystrophy: lipoatrophy, but not central fat gain, is an antiretroviral adverse drug reaction. PLoS One.

[18] Stanley TL, Grinspoon SK (2012). Body composition and metabolic changes in HIV-infected patients. J Infect Dis.

[19] Peer N, Steyn K, Lombard C, Lambert EV, Vythilingum B, Levitt NS (2012). Rising diabetes prevalence among urban-dwelling black South Africans. PLoS One.

[20] Malaza A, Mossong J, Bärnighausen T, Newell M (2012). Hypertension and obesity in adults living in a high HIV prevalence rural area in South Africa. PLoS One.

[21] Semeere AS, Sempa J, Lwanga I, Parkes-Ratanshi R, Kambugu A (2014). Hypertension and associated risk factors in individuals infected with HIV on antiretroviral therapy at an urban HIV clinic in Uganda. The Lancet Global Health.

[22] Manuthu EM, Joshi M, Lule G, Karari E (2008). Prevalence of dyslipidemia and dysglycaemia in HIV infected patients. East Afr Med J.

[23] Zinn RJ, Serrurier C, Takuva S, Sanne I, Menezes CN (2013). HIV-associated lipodystrophy in South Africa: the impact on the patient and the impact on the plastic surgeon. J Plast Reconstr Aesthet Surg.

[24] Department of Health (2013) The South African Antiretroviral Treatment Guidelines

[25] Carr A (2003). An objective case definition of lipodystrophy in HIV-infected adults: a case-control study. Lancet.

[26] Lichtenstein KA, Ward DJ, Moorman AC, Delaney KM, Young B, Palella FJ (2001). Clinical assessment of HIV-associated lipodystrophy in an ambulatory population. AIDS.

[27] American Diabetes Association (2010). Diagnosis and classification of diabetes mellitus. Diabetes Care.

